# Motor synchronization and impulsivity in pediatric borderline personality disorder with and without attention-deficit hyperactivity disorder: an eye-tracking study of saccade, blink and pupil behavior

**DOI:** 10.3389/fnins.2023.1179765

**Published:** 2023-06-22

**Authors:** Olivia G. Calancie, Ashley C. Parr, Don C. Brien, Jeff Huang, Isabell C. Pitigoi, Brian C. Coe, Linda Booij, Sarosh Khalid-Khan, Douglas P. Munoz

**Affiliations:** ^1^Queen’s Eye Movement Lab, Centre for Neuroscience Studies, Queen’s University, Kingston, ON, Canada; ^2^Department of Psychiatry, University of Pittsburgh, Pittsburgh, PA, United States; ^3^Department of Psychiatry, McGill University, Montreal, QC, Canada; ^4^Research Centre and Eating Disorders Continuum, Douglas Mental Health University Institute, Montreal, QC, Canada; ^5^Divison of Child and Youth Psychiatry, Department of Psychiatry, School of Medicine, Queen’s University, Kingston, ON, Canada

**Keywords:** predict saccade, response inhibition, eye movement, metronome, psychotropic medication, arousal, psychiatric disease

## Abstract

Shifting motor actions from reflexively reacting to an environmental stimulus to predicting it allows for smooth synchronization of behavior with the outside world. This shift relies on the identification of patterns within the stimulus – knowing when a stimulus is predictable and when it is not – and launching motor actions accordingly. Failure to identify predictable stimuli results in movement delays whereas failure to recognize unpredictable stimuli results in early movements with incomplete information that can result in errors. Here we used a metronome task, combined with video-based eye-tracking, to quantify temporal predictive learning and performance to regularly paced visual targets at 5 different interstimulus intervals (ISIs). We compared these results to the random task where the timing of the target was randomized at each target step. We completed these tasks in female pediatric psychiatry patients (age range: 11–18 years) with borderline personality disorder (BPD) symptoms, with (*n* = 22) and without (*n* = 23) a comorbid attention-deficit hyperactivity disorder (ADHD) diagnosis, against controls (*n* = 35). Compared to controls, BPD and ADHD/BPD cohorts showed no differences in their predictive saccade performance to metronome targets, however, when targets were random ADHD/BPD participants made significantly more anticipatory saccades (i.e., guesses of target arrival). The ADHD/BPD group also significantly increased their blink rate and pupil size when initiating movements to predictable versus unpredictable targets, likely a reflection of increased neural effort for motor synchronization. BPD and ADHD/BPD groups showed increased sympathetic tone evidenced by larger pupil sizes than controls. Together, these results support normal temporal motor prediction in BPD with and without ADHD, reduced response inhibition in BPD with comorbid ADHD, and increased pupil sizes in BPD patients. Further these results emphasize the importance of controlling for comorbid ADHD when querying BPD pathology.

## 1. Introduction

Borderline personality disorder (BPD) affects 1–2% of the population and represents a significant portion (15–30%) of patients in psychiatric clinics and inpatient hospitals (Gunderson et al., 2018). Individuals with BPD are highly susceptible to addiction, disability, incarceration, and death by suicide (Leichsenring et al., 2011). The Diagnostic and Statistical Manual of Mental Disorders (DSM-V) defines BPD based on nine distinct traits, requiring individuals to exhibit at least five of these traits for a diagnosis (American Psychiatric Association, 2022). These traits encompass emotional instability, efforts to avoid abandonment, identity disturbance, chronic feelings of emptiness, difficulty controlling anger, patterns of interpersonal conflict, recurrent suicidal behavior, stress-related paranoid ideation or dissociation, and impulsive behavior. As BPD symptoms typically emerge during adolescence (Wright et al., 2016), it is crucial to characterize the biology of the disease during this stage to facilitate early identification and therapeutic intervention.

Among the various techniques available for investigating brain-based behavior, video-based eye-tracking has emerged as a powerful non-invasive tool that can assess different levels of the nervous system (McDowell et al., 2008; Eckstein et al., 2017). Firstly, vision provides valuable insights into the integrity of various structures such as the retina, optic nerves, brainstem, thalamic lateral geniculate nuclei, and visual cortex. Secondly, the coordination of rapid eye movements, known as saccades, for visual scene exploration involves neural signaling from widespread areas of the brain, including the cortex, subcortical nuclei, brainstem, and cerebellum (Liversedge et al., 2011). In fact, more than 50% of cortical surface is dedicated to visual processing. Thirdly, blinks, in addition to maintaining the health of the anterior surface of the eye, have been found to vary with cognitive demand and are sensitive to disruptions in dopaminergic neurotransmitter signaling, as seen in conditions like Parkinson’s disease and Schizophrenia (Basso et al., 1996; Swarztrauber and Fujikawa, 1998; Taylor et al., 1999; Schmahmann, 2000; Agostino et al., 2008; Cruz et al., 2011). Fourthly, the constriction and dilation of pupils, regulated by the sphincter and dilator muscles respectively, reflect the tone of the parasympathetic and sympathetic nervous systems (Kandel et al., 2000). By precisely quantifying eye behavior in terms of saccades, blinks, and pupil responses, it is possible to gain insights into the underlying function of the nervous system, ranging from neurotransmitter levels to neural circuitry. Indeed, the extensive body of eye-tracking literature demonstrates how eye behavior changes during human development (Luna et al., 2008; Calancie et al., 2022; Yep et al., 2022), aging (Peltsch et al., 2011; Yep et al., 2022), psychiatric pathology (Hutton and Ettinger, 2006; Gooding and Basso, 2008; Rommelse et al., 2008; Thakkar et al., 2011; Huang et al., 2022), and neurologic disease (Brien et al., 2023; Riek et al., 2023).

Previous eye-tracking studies have provided valuable insights into the eye behavior of individuals with borderline personality disorder (Grootens et al., 2008; Rentrop et al., 2008; Jacob et al., 2010; Bertsch et al., 2017; Kaiser et al., 2019; Bortolla et al., 2020; Seitz et al., 2021; Parr et al., 2022), with all but one (Parr et al., 2022) describing BPD in adults. These eye-tracking studies have reported an increased incidence of anticipatory saccades in BPD (Grootens et al., 2008; Parr et al., 2022), which are characterized by initiating eye movements before the minimum required time for visual target processing and subsequent saccade execution. Anticipatory saccades can be viewed as indicators of irregular waiting impulsivity, reflecting the ability to withhold actions to gather sufficient information for making accurate choices (Dalley and Robbins, 2017). This behavior has been associated with diminished prefrontal cortex activity and has been observed in other psychiatric pathologies (Pierrot-deseilligny et al., 1991; Gaymard et al., 1998; Ross et al., 1998; Walker et al., 1998; Carr et al., 2006; Yep et al., 2018). However, the extent to which this finding in BPD is influenced by presence of comorbid psychopathology, such as ADHD, remains unclear. Furthermore, studies have shown that individuals with BPD exhibit faster saccade reaction times to emotionally valent stimuli compared to controls, suggesting an attentional bias toward negative emotional stimuli in particular, consistent with the reliable findings of limbic system hyperactivity (Schulze et al., 2016; Bertsch et al., 2017; Bortolla et al., 2020; Seitz et al., 2021).

Despite these studies, there is limited understanding of other basic saccade parameters, such as peak velocity and amplitude, in individuals with BPD, as most previous eye-tracking studies have focused solely on saccade reaction time. Consequently, the functioning of the reflexive saccade system in BPD remains poorly understood. Similarly, blink behaviors in BPD have received a paucity of research attention. Although two previous studies using startle response paradigms found no differences in induced blink responses between non-medicated BPD patients and controls (Herpertz and Koetting, 2005; Limberg et al., 2011), there is a dearth of studies examining baseline blink rate or the modulation of blink rate by different task sets in BPD. Considering that antipsychotic medication is commonly prescribed for managing BPD and affects dopaminergic and other signaling pathways (Jongkees and Colzato, 2016), it is plausible that blink rates may be altered in individuals with BPD.

Pupillary analysis has been explored in one study of BPD, which revealed increased pupillary dilation magnitude in adolescent females with BPD when exposed to maternal criticism (Scott et al., 2017). However, it remains unknown whether pupil size is larger than that of matched controls under emotionally neutral conditions. Given the heightened incidences of aggression, self-harm, and impulsivity in individuals with BPD (Stepp et al., 2012; Gunderson et al., 2018), characteristic of the fight or flight response, it is hypothesized that sympathetic nervous system tone is elevated in BPD patients, leading to increased pupil diameters compared to controls at rest.

To further investigate eye behavior in BPD, the present study collected video-based eye-tracking data from adolescent patients with BPD and age-matched controls. Two oculomotor tasks were performed, involving the movement of eyes toward visual targets with different temporal predictabilities. In the metronome task, participants were instructed to move their eyes in time with a target that alternated between two fixed locations at a fixed interstimulus interval (ISI; 5 ISIs were tested ranging from 500–1,500 ms). The random task was identical to the metronome task except the timing of the target was randomized at each step, making the target’s location predictable but its timing unpredictable. In the metronome task, successful prediction of targets requires maintenance of the ISI and the production of voluntary saccades that match the timing of target arrival. In the random task, however, participants need to only reflexively react to targets given that their timing was unknown. The tasks aimed to evaluate variations in blink and pupil responses associated with different task difficulties. As BPD is frequently comorbid with ADHD (with an incidence of approximately 50%) (Philipsen et al., 2008; Fossati et al., 2014), and previous research has shown distinct behaviors in individuals with comorbid ADHD and BPD, the data of individuals with BPD and comorbid ADHD (referred to as ‘ADHD/BPD’) were analyzed separately. Additionally, considering that most pediatric BPD patients receiving medical care at our tertiary care hospital were female, and previous BPD studies have supported gender-based differences in BPD behavior, we specifically recruited female participants for this study (McCormick et al., 2007; Herpertz et al., 2017).

## 2. Materials and methods

### 2.1. Participants and study recruitment

The research protocol was reviewed and approved by Queen’s University Faculty of Health Sciences (protocol ID: PHYS-007-97). We recruited females aged 11–18 years (*Mean* age = 15.8 ± *Standard Deviation* = 1.6 years), inclusive for study participation. Child and adolescent psychiatrist co-author SKK recruited pediatric psychiatry participants from Hotel Dieu Hospital Child and Youth Mental Health’s Dialectical Behavioral Therapy outpatient clinic which provides psychiatric care for pediatric patients with signs of BPD. Patients attending this clinic have high health care utilization (e.g., multiple emergency room visits for suicide attempts and frequent resulting hospitalizations). Those who were interested in study participation completed an in-person interview for BPD symptomology, known as the Structured Clinical Interview for DSM-5 Diagnosis for Borderline Personality Disorder (SCID-PD-5). The interview was delivered by a graduate student who received SCID-PD-5 training and supervision by SKK. Patients who met criteria for BPD on the SCID-PD-5 (i.e., >5 responses with a score of 2) were invited to participate in the research study and are referred to the ‘BPD’ participant group moving forward. Members of the BPD participant group who had a pre-existing comorbid diagnosis of ADHD diagnosed by a child and adolescent psychiatrist were analyzed separately as the ‘ADHD/BPD’ group. Participants in the BPD and ADHD/BPD groups were not asked to interrupt their medication regimen on the day of testing and any medications that the patients were taking were documented. Participants had normal or corrected-to-normal vision and were free of ocular conditions. Control participants were recruited via study flyers advertised in the local newspaper and university campuses. Control participants were required to meet the following criteria for study inclusion: (1) absence of neurological, ocular, or psychiatric diagnoses; (2) not taking psychotropic medications; and (3) normal or corrected-to-normal vision.

Participants aged ≥17 years old provided written consent. Participants aged 11–16 years provided their oral assent and written consent was obtained by a legal guardian. Participants were compensated for their time at a rate of $20/h. Clinical participants filled out self-report scales to provide measures of BPD and ADHD symptomology. Based on previous eye-tracking studies in BPD and ADHD cohorts (Gould et al., 2001; Grootens et al., 2008; Hakvoort Schwerdtfeger et al., 2013; Parr et al., 2022), sample sizes for adequate statistical power was estimated with the following parameters: ANOVA design (3 groups: BPD, ADHD/BPD, and control), 0.05 alpha level, 0.8 power, and 0.5 medium effect size. It was determined that a sample size of 64 resulted in a statistical test power of 0.805, resulting in 21.3 participants per group.

### 2.2. Eye-tracking set-up and task paradigms

#### 2.2.1. Eye-tracking set-up

Eye-tracking was completed in a black-out room with participants seated and their head position stabilized by a fixed head mount and chin rest. Participants were seated 60 cm away from a 17-inch LCD iiYama Prolite monitor with a viewing angle of 32 × 26 degrees. The LCD monitor has a refresh rate of 60-Hz with a screen resolution of 1,280 × 1,024 pixels. Monocular eye position was recorded at a sampling rate of 500 Hz with an infrared video-based eye-tracker (Eyelink 1000 Plus, SR Research Ltd., ON, Canada). Prior to completing the experimental tasks, participants performed a 9-point calibration and validation procedure. Accuracy of eye position from target was required to be within 1.5° for collection of eye-tracking data.

#### 2.2.2. Experimental task paradigms

**Figure 1:**
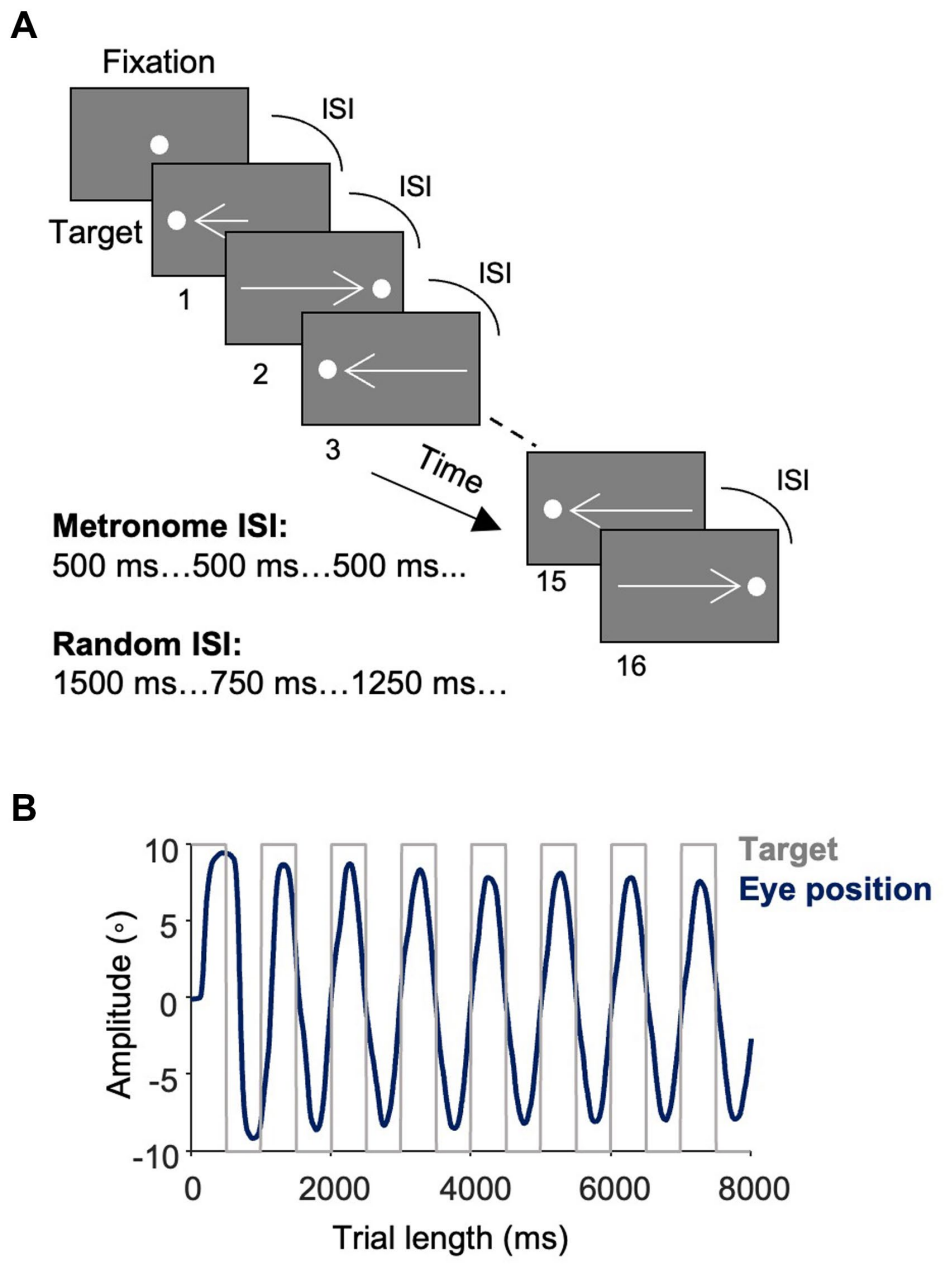
Task design and saccade responses. **(A)** Metronome task. Trials started with a central fixation point (FP) with a random offset between 1,000–1,500 ms. A white target spot appeared at 10° eccentricity at the time of FP offset and alternated between the right and left side of the screen at a fixed interstimulus interval (ISI) for 16 target steps. Participants were instructed to move their eyes in time with the target. Five ISI conditions were used: 500 ms, 750 ms, 1,000 ms, 1,250 ms, 1,500 ms with each ISI having 6 trial blocks of 16 target steps. Different trial blocks of ISI conditions were presented pseudorandomly. In the random task, the ISI varied with each target step such that the location of the targets remained predictable, but the timing of their onset was unpredictable. **(B)** Eye position. The average of study participants’ traces of eye positions (dark blue) toward the square-wave target (light gray) in the 500 ms condition of the metronome task.

Two eye-tracking tasks (Figure 1A) were completed by each participant, the *metronome task* and *random task*. In the metronome task, participants were cued with a central fixation point (FP; 0.5° in diameter and 44 cd/m^2^ in luminance) with a random offset between 1,000–1,500 ms. A target then appeared on the right-side of the screen, 10 degrees away from central FP. The target proceeded to step between the right and left side of the screen for 16-target steps. Thereby the first target step was 10 degrees away from the central FP, with the following target steps were 20 degrees in the opposite direction from the previous target step (Figure 1B). The timing of the ISI was held constant for each of the 16 target steps – with 5 different ISI durations tested: 500 ms (i.e., 2 target steps per second), 750 ms, 1,000 ms, 1,250 ms and 1,500 ms. Thus, the location and timing of target appearance was predictable. Participants completed 6 blocks of each ISI, yielding 96 total target steps per ISI. ISI conditions were arranged with pseudorandom order so that all participants were exposed to the same order of ISI blocks. Participants were instructed to try and match the timing of their saccade with the appearance of the target.

The random task was identical to the metronome task except that the ISI changed with each target step, such that the direction of the target remained predictable, however, the timing of its appearance was random. The same 5 ISIs were used for target steps and a pseudorandom order was used, such that participants were never cued with more than two identical ISIs back-to-back. Like the metronome task, participants completed a total of 30 experimental blocks of 16 target steps. In both tasks, participants performed a re-calibration and validation procedure every 5 blocks to ensure accurate eye-tracking during the duration of the recording. The order of tasks was randomized so that some participants completed the metronome task first and others did the random task first. Together, the metronome and random tasks took about 25 min to complete.

#### 2.2.3. Self-report clinical questionnaires

Self-report measures of impulsivity, BPD symptoms and suicidality were obtained by clinical participants prior to eye-tracking. Impulsivity was assessed using the Barratt Impulsivity Scale (BIS), a well-validated tool that provides a measure of impulsivity as a total score and domains of impulsivity as subscale scores (Barratt, 1959; Moeller et al., 2001). Subscales include Attention, Motor, Cognitive Instability, Perseverance, Self-control, and Cognitive Complexity. BPD symptom severity was measured with the Borderline Symptom List 23 (BSL) which asks over the last week how often individuals felt statements such as, ‘I did not trust other people’, ‘My mood rapidly cycled in terms of anxiety, anger, and depression’, and ‘Criticism has a devastating effect on me’ (Bohus et al., 2009). Suicidality was assessed with the Suicide Behaviors Questionnaire – Revised (SBQ), which asks statements such as, ‘Have you ever thought about or attempted to kill yourself?’ and ‘How often have you thought about killing yourself in the past year?’ (Osman et al., 2001). A cut-off score of ≥7 has a sensitivity of 93% and specificity of 95% of detecting individuals at suicide risk.

### 2.3. Behavior detection and analysis

#### 2.3.1. Saccade detection

Data from Eyelink 1,000 Plus were analyzed offline using Matlab version R2022 (MathWorks). Saccades were labeled when the instantaneous velocity of eye position in the x and y plane was greater than 2 standard deviations above the mean fixation velocity (defined as <50°/s) for at least five continuous data points.

#### 2.3.2. Eye-tracking parameters

##### 2.3.2.1. Saccade

**Figure 2:**
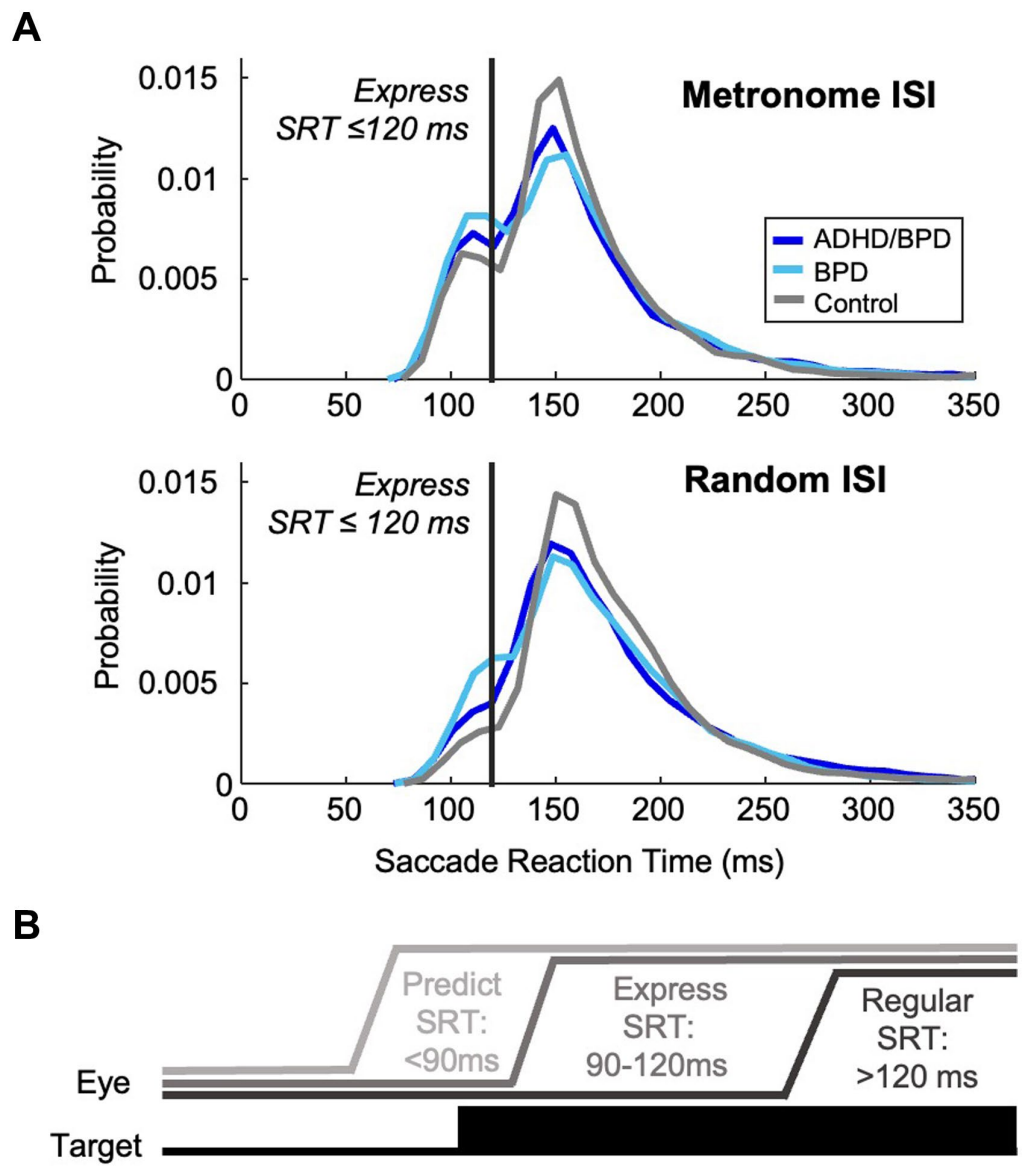
Characterization of the express saccade epoch. **(A)** Probability density estimate of participants’ visually guided saccades with reaction times >90 ms in the metronome and random task. Bimodality coefficient analysis of these reactive saccades supported a bimodal distribution of saccade reaction times (skewness = 6.50; kurtosis = 71.7), supportive of distinct populations of saccades, demonstrating express and regular latencies. The deflection points of the distribution of SRTs occurred at ~120 ms in both task paradigms. Express saccades were thereby categorized as those with SRTs within 90–120 ms, inclusive, and regular-latency saccades were defined as those with RTs > 120 ms. **(B)** Schematic of eye position relative to target by saccade type.

Metrics of saccade data included saccade reaction time (SRT), amplitude (°), and peak velocity (°/s). SRT was calculated by subtracting the time of the start of a saccade from target appearance. Saccades were defined as *predict*, *express* or *regular* according to SRT. Predict saccades are generated before the minimum amount of time needed for neural signaling of the visual target has elapsed and a saccade motor command to said target is generated. Previous work showed that when generating a saccade to one of two potential target locations, saccades with RTs > 90 ms had an accuracy above 95%, whereas those initiated with RTs < 90 ms had a chance accuracy of 50%, indicating the lower limit for SRTs to visual stimuli as 90 ms (Munoz et al., 1998). Saccades made before 90 ms are considered a *guess* to a potential target location and are internally generated. Express saccades occur when the incoming visual transient signal combines with a saccade motor command, causing ultra-fast saccades to targets. The upper epoch of the express saccade window has been shown to vary with experimental conditions (i.e., target luminance, presence of a fixation gap, and target eccentricity) (Weber et al., 1992; Paré and Munoz, 1996; Dorris and Munoz, 1998; Marino and Munoz, 2009; Marino et al., 2015). We thus quantified the express epoch according to the distribution of SRTs in the metronome and random tasks. Bimodal coefficient testing was performed to identify the change in SRT distribution between express and regular saccade latencies (when the visual transient signal does not combine with a saccade motor command). For both metronome and random tasks, the deflection in SRT distribution between the two saccade populations was observed at ~120 ms (Figure 2A). Accordingly, the express saccade epoch for this paper was defined as 90–120 ms, inclusive (Figure 2B), in agreement with a previous study with the same experimental tasks (Calancie et al., 2022). Regular latency saccades were defined as those with SRTs >120 ms. Percentage of trials with predict, express and regular saccades to targets was calculated per subject in the 5 metronome ISI conditions and in the random task and averaged within groups to allow for group comparison.

Saccade amplitude to target was measured in degrees with a maximum eye position error of ±1.5° away from target location. Peak saccade velocity was calculated for each saccade with its maximum set to 1,000°/s, as saccades made above this threshold are due to signal noise and do not reflect true saccades. The main sequence of saccades was calculated for individual subjects using square root models of amplitude and peak velocity, which has been shown to be a robust model for main sequence characterization of saccades with amplitudes between 5–20° (Gibaldi and Sabatini, 2021). Individual main sequence models were then averaged for each group and model coefficients and 95% CIs are reported.

##### 2.3.2.2. Blink

During eye-tracking there are periods when the eye position signal is absent, known as eye loss – some of which are caused by blinks. To separate eye loss due to *blinks* from eye loss due to poor video-based tracking or not following the instructions of the experimental task, we plotted the durations of eye loss. As supported by previous blink literature (Caffier et al., 2003; Betke and Chau, 2005), we observed a consistent profile of eye loss durations between 50–300 ms characteristic of blinks and therefore used this duration limit for blink classification and subsequent analysis. Blink rates and blink reaction times relative to target appearance were computed for the experimental paradigms.

In the metronome task, blink probability was analyzed for each individual subject from −1,000 ms to +1,000 ms relative to target appearance. A logical array was computed for individual subjects at each data collection point across the trial length (i.e., 500 Hz sampling rate aka every 2 ms). The 2 ms timeslot was represented with a 0 when the participant was not currently in a blink or a 1 when they were blinking. Individuals’ logical arrays were then averaged across the 480 metronome trials to create an average of blink probability per subject. Re-sampling via bootstrapping with replacement was performed to generate blink probability means and confidence intervals (CIs). Difference scores were then calculated between groups to allow for statistical comparison (i.e., re-sampled mean of ADHD/BPD blink probability – re-sampled mean of BPD blink probability). 95% CIs were computed for each difference score. If the 95% CI for the difference score did not include 0, the difference scores were considered significantly different from one another, supporting that blink probability varied by group at that time. We report periods of significant difference scores across the trial epoch for each group comparison as well as the corresponding standardized Cohen d effect sizes. Smoothing splines were fit to the blink probability and Cohen d effect size data using a fraction of the total number of data points approach (fraction = 0.05; the smoothed value reflected 100 ms of trial duration).

##### 2.3.2.3. Pupil

Pupil size was recorded throughout the duration of the eye-tracking tasks to determine eye position. To provide a measure of average pupil size, indicative of autonomic tone, recordings were averaged over 200 ms following a saccade to target. This epoch was selected to avoid the pupillary light reflex triggered by target luminance which is known to take into effect >200 ms after stimulus appearance (Ellis, 1981; Wang et al., 2018). By analyzing pupil size post-saccade on a trial-by-trial level, its size could be subsequently compared by saccade type (i.e., predict, express, regular) as well as participant group. Pupil traces were included for analysis if they met the following criteria: (1) fixation occurred for a minimum of 200 ms; (2) the timing of fixation onset exceeded 100 ms between fixation and the next target appearance; (3) pupil velocity was within −5,000°/s to 5,000°/s; and (4) the pupil trace was free of any blinks or eye loss.

### 2.4. Statistical testing and reporting

Eye behavior data analyzed were saccade metrics (i.e., SRT, velocity, amplitude, main sequence), % predict, express, and regular saccades to metronome and random ISI targets, blinks (i.e., blink rate, blink reaction time to target, blink probability across trial length) and pupil size. Independent variables were group membership (ADHD/BPD, BPD, and control), task (metronome; random), and ISI (5 different ISIs were tested in the metronome task). Before group comparison analysis, data were tested for normalcy using the Shapiro–Wilk test. If eye-tracking data did not meet the Shapiro–Wilk’s test for normality, non-parametric tests were performed, and mean ranks were reported. Group differences in eye behavior and task performance were assessed using the Kruskal-Wallis test for non-parametric data or ANOVA for parametric data. Analysis of group differences are based on the group mean of individual means of eye behavior variables. Correlation analyses were performed for clinical questionnaire data and eye behaviors using Spearman rank test or Pearson correlation depending on the data skewness. Main effects were followed-up with *post-hoc t*-tests using the Bonferroni correction to protect against type I errors (i.e., 0.05/number of eye behavior variables = corrected alpha) and effect sizes are reported as an eta-squared or Cohen’s d. Based on previous papers that performed the metronome and random tasks in neuropsychiatric patients, we expected to observe a small to medium effect size for differences in % predict saccades to alternating targets between groups (Thakkar and Rolfs, 2019; Vaca-Palomares et al., 2019; Deravet et al., 2021). Due to the novel nature of blink and pupil analysis in BPD and ADHD/BPD participants during a saccade task, we did not have a prior expectation of the magnitude of effect sizes for blink and pupil variables.

## 3. Results

### 3.1. Participants

Table 1 shows the demographic data of study participants. 23 BPD, 22 ADHD/BPD and 35 control participants completed the research study and were included for analysis. Participants were on average 15–16 years of age and all participants were Female. Clinical participants were not asked to interrupt their medication regimen and their prescribed psychotropic drugs are reported in Table 1. Clinical participants filled out self-report questionnaires on BPD and ADHD symptomology in addition to eye-tracking testing (Table 2). Scores of psychiatric symptomology did not differ among BPD and ADHD/BPD participants.

**Table 1:** Summary of demographic features and psychotropic medication prescriptions to clinical participants.

	ADHD/BPD mean (SD)	BPD mean (SD)	Control mean (SD)
Sample size (*N*)	22 F	23 F	35 F
Age (range = 11–18 years)	16.32 (1.55)	16.42 (1.48)	15.26 (1.43)
Medication Classes			
Stimulants (% of participants)	9 (40.9%)	4 (17.4%)	0
SSRIs (% of participants)	12 (54.5%)	17 (73.9%)	0
Second-generation antipsychotics (% of participants)	9 (40.9%)	9 (39.1%)	0

Clinical participants stayed on their medication regimen on the day of eye-tracking testing and reported the psychotropic agents that they were prescribed. Psychotropic drugs were classified according to whether they were among the stimulant, SSRI, or SGA medication class. Medication data are reported as the number (percentage) of participants within the given clinical group on that class of medication. Three psychiatric participants were not taking psychotropic medication, 22 were taking a drug from 1 psychotropic class, 15 from 2 classes, and 4 from 3 classes. Medication data was unavailable for 1 participant. No controls reported taking psychotropic medications on the day of testing. F, Female; SSRI, Selective Serotonin Reuptake Inhibitors.

**Table 2:** Self-report scores of impulsive and borderline personality disorder symptoms among clinical participants.

	ADHD/BPD mean (SD)	BPD mean (SD)	*T*-statistic	*p*
Impulsivity (BIS)				
Total	81.81 (10.56)	75.36 (8.29)	3.24	0.08
Motor	19.00 (4.02)	16.45 (3.42)	0.36	0.55
Cognitive instability	8.71 (1.71)	8.45 (2.02)	0.64	0.43
Attention	14.76 (2.98)	13.00 (2.12)	0.84	0.37
Self-control	17.14 (3.98)	15.91 (3.50)	0.55	0.46
Cognitive complexity	13.05 (2.80)	12.95 (2.03)	1.88	0.18
Perseverance	9.14 (2.01)	8.59 (2.20)	0.92	0.34
BPD Symptoms (BSL)	44.43 (25.09)	53.05 (23.98)	−1.15	0.26
Suicidality (SBQ)	13.63 (2.91)	13.85 (3.22)	0.22	0.83

ADHD/BPD and BPD patients did not significantly differ in their self-report scores in the BIS (Barratt Impulsivity Scale), BSL (Borderline Symptom List-23), or SBQ (Suicidal Behaviors Questionnaire).

### 3.2. Saccade behavior

#### 3.2.1. Saccade main sequence and reaction time

The saccade main sequence was estimated per subject in the metronome task using saccade data from the 5 ISI conditions pooled together and then averaged for participant groups (Supplementary Figure S1). As expected, there was a significant effect of saccade type on the main sequence (*F* [2,234] = 28.5; *p* = 8.38e-12, η^2^ = 0.196), with a significantly lower main sequence observed for predict saccades (Mean model coefficient = 96.6, *SD* = 1.80) versus express (*M* = 112.3, *SD* = 17.2; *p* < 5.117e-10) and regular (*M* = 111.5, *SD* = 16.6; *p* < 2.75e-9). Main sequence fits for express and regular saccades did not differ. Main sequence model coefficients did not vary by participant group (*p* = 0.332); see Supplementary Figure S1 for model coefficients and 95% CIs. Average SRTs are plotted for each target step in Supplementary Figure S2 in the random and metronome task. In the metronome task, participants generally made saccades with RTs < 90 ms (marked by a horizontal gray line) by target steps 3–4. SRTs to randomized targets were on average predictive in ADHD/BPD participants compared to reactive in BPD and control groups.

#### 3.2.2. Temporal prediction

The percentage of predict, express or regular saccades did not differ among groups in the metronome task (see Table 3). In general, study participants produced 50–70% of saccades in anticipation of the target appearance, with fewer predict saccades observed in the slower pacing conditions (i.e., 1,250 ms and 1,500 ms ISI conditions). The percentage of predict saccades was consistently higher in the BPD group compared to ADHD/BPD and controls, however, this effect was non-significant. In the random task, the percentage of predict saccades significantly varied by group [χ^2^ (2) = 11.13; *p* = 0.004; η^2^ = 0.148; with a *Mean* rank of 49.64 for ADHD/BPD, 43.93 for BPD and 30.07 for controls]. *Post-hoc* tests revealed that ADHD/BPD participants made significantly more predict saccades than controls (*p* = 0.005; no other groups differed). Express saccades varied by group in the random task [χ^2^ (2) = 9.93; *p* = 0.007; *Mean* rank of 42.34 for ADHD/BPD, 49.95 for BPD and 30.90 for controls], with BPD subjects generating more express saccades than controls (*p* = 0.006). Additionally, a main effect of group on the percentage of regular saccades was found in the random task, *χ*^2^ (2) = 14.86; *p* = 0.001; *Mean* rank of 30.14 for ADHD/BPD, 31.50 for BPD and 50.74 for controls. Both psychiatric cohorts, ADHD/BPD (*p* = 0.003) and BPD (*p* = 0.006), made significantly fewer regular saccades in the random task versus controls.

**Table 3:** Percentage of predictive saccades toward randomized and predictable ISI targets.

	ADHD/BPD mean ± SD	BPD mean ± SD	Control mean ± SD	Chi-square	*p*
% of Predict saccades					
Random ISI	25.12 ± 16.78%	18.48 ± 10.69%	12.68 ± 10.55%	11.13	0.004
500 ms ISI	59.61 ± 20.62%	68.23 ± 17.19%	65.20 ± 18.86%	2.75	0.25
750 ms ISI	61.82 ± 17.94%	69.59 ± 17.06%	67.59 ± 19.45%	3.33	0.19
1,000 ms ISI	56.18 ± 19.64%	60.12 ± 19.14%	56.42 ± 18.95%	0.66	0.72
1,250 ms ISI	49.71 ± 18.34%	50.56 ± 19.50%	45.12 ± 19.62%	1.44	0.49
1,500 ms ISI	41.12 ± 19.51%	43.78 ± 18.63%	39.50 ± 19.30%	1.0	0.58

A main effect of participant group x % of predictive saccades was observed in the random task. Post-hoc *t*-tests showed that the ADHD/BPD group made significantly more predictive saccades compared to controls. Participant groups did not significantly differ in their percentage of predictive saccades toward metronome targets in any of the ISI conditions. As the ISI lengthened, % of predictive saccades decreased for all groups.

The target step for when individuals made their first predict saccade to regularly paced targets was analyzed to estimate speed of temporal predictive learning among groups. Kruskal-Wallis tests were performed with a Bonferroni correction applied for multiple comparisons (0.05/5 = 0.01 corrected alpha). Results found no group effect for four of the five ISIs. A significant effect was noted for the 1,000 ms ISI condition (KW test statistic = 9.917, *p* = 0.007) with a *post-hoc* significant difference between controls (*Median* = 4.00 ± *Standard Deviation* = 2.17) and BPD (3.00 ± 1.82; *p* = 0.002). On average, BPD participants launched their first predict saccade one target step earlier than controls (i.e., target step 3 versus 4). Accuracy of temporal prediction to periodic targets was calculated based on the time gap between the end of the saccade and target appearance in milliseconds. There was no effect of group or ISI on temporal prediction accuracy (*F* = 2.604, *p* = 0.075; *F* = 1.675, *p* = 0.155, respectively). On average, participants ended their saccade ~100–200 ms prior to target appearance (Supplementary Figure S3).

### 3.3. Blink behavior

#### 3.3.1. Blink duration

**Figure 3:**
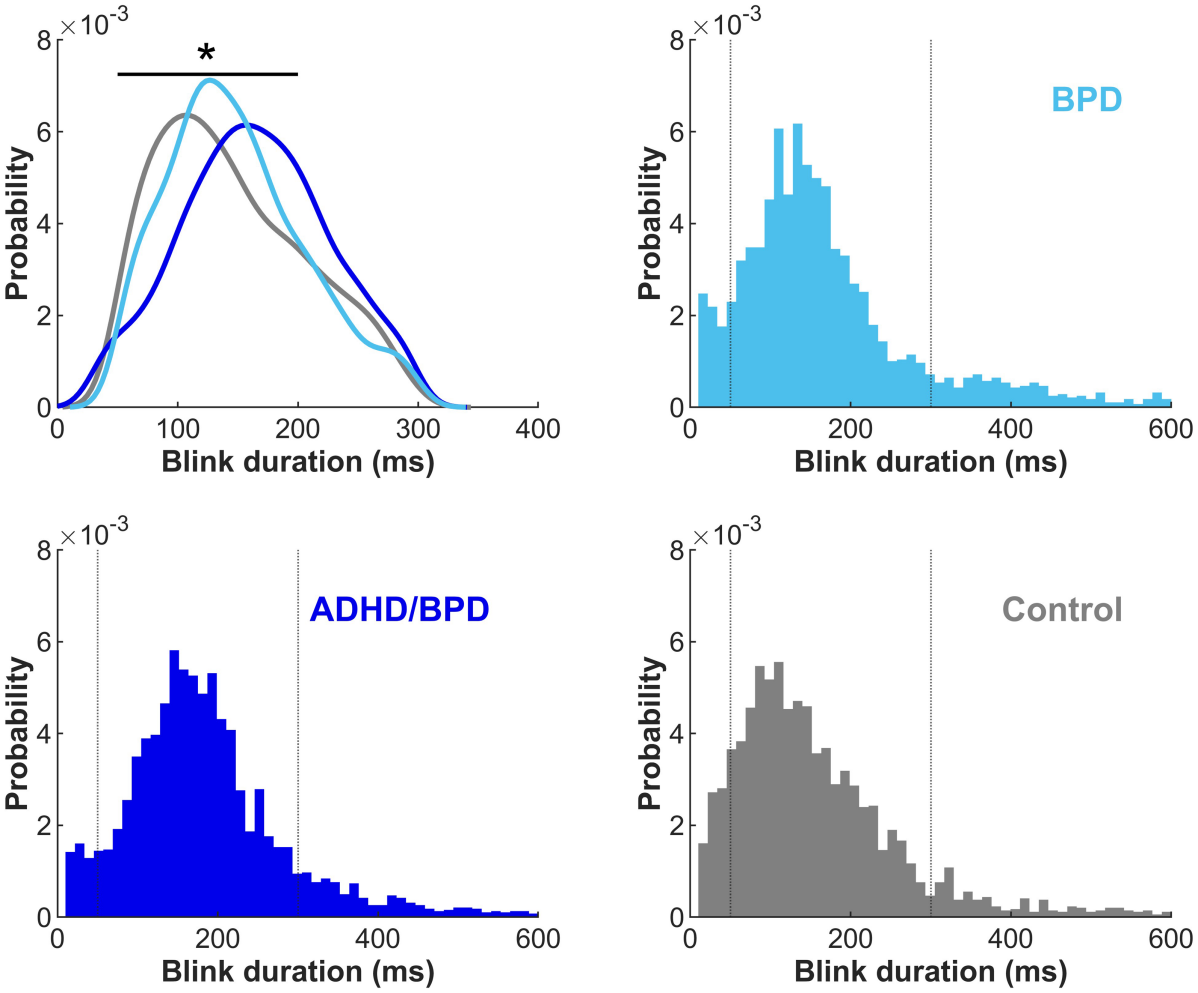
Duration of blinks by participant group in the metronome and random tasks combined. Kernel density estimate for eye loss durations between 50–300 ms are plotted by participant group in the upper left panel. The remaining three panels show eye loss durations with a range of 10–600 ms and a bin width of 50 ms. Dotted vertical lines represent the eye loss duration cut-off (50–300 ms) for blink analysis inclusion. Duration of blinks significantly varied by participant group, with ADHD/BPD participants making significantly longer blinks than control participants (*p* < 0.01).

Durations of lost eye-tracking were plotted as histograms to identify signal loss that was characteristic of blinks (Figure 3). In agreement with previous literature, our data supported increased periods of eye loss between 50–300 ms, characteristic of blink durations (Caffier et al., 2003; Betke and Chau, 2005). Eye loss within this range was labeled as blinks for subsequent analyses, see dotted vertical lines denoting this range in Figure 3. A main effect of group on blink duration was observed (*F* (2,75) = 5.64; *p* = 0.0052), with *post-hoc* statistics revealing that individuals with ADHD/BPD made on average significantly longer blink durations (*Mean* = 167.3 ms ± *SD* = 28.3) compared to controls (139.9 ms ± 32.0; *p* = 0.0054) but not compared to BPD (142.925 ms ± 30.914; *p* = 0.0277; failed Bonferroni correction for multiple comparisons).

#### 3.3.2. Blink rate by diagnosis and psychotropic medication

**Figure 4:**
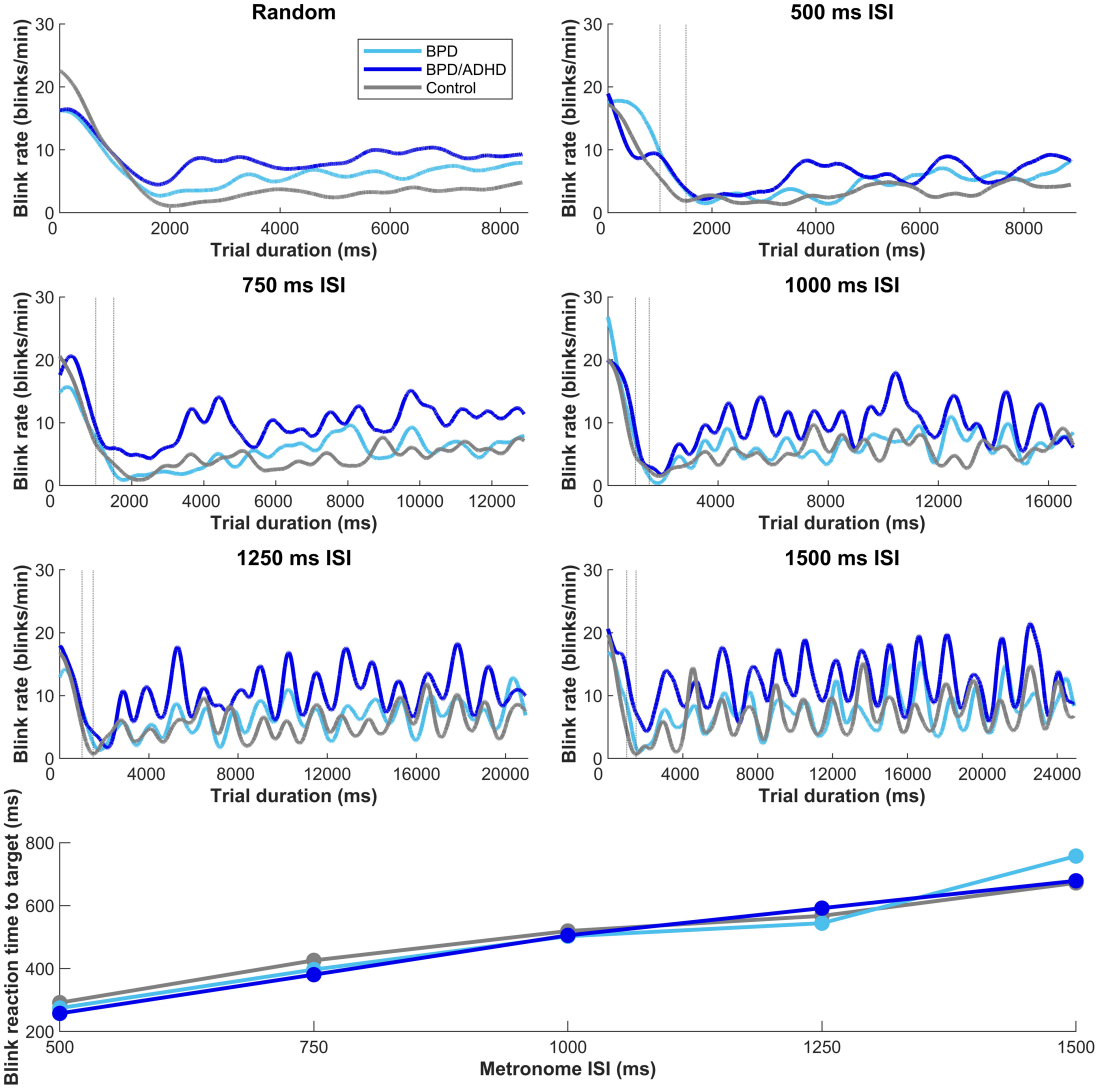
Moving average of blink rate in the metronome and random task across trial duration. Dashed vertical lines represent the random offset between 1,000–1,500 ms when the central fixation point at the start of trial disappeared. A main effect of blink rate by participant group was observed in the metronome task, with *t*-tests revealing higher blink rates in ADHD/BPD than BPD (*p* = 0.002) and controls (*p* < 0.001). Mean blink rates displayed a sinusoidal structure after the first 1–3 target steps, with the subsequent number of peaks corresponding to the number of target steps. No main effect of blink rate x group was observed when the ISI of targets was randomized (top left panel). In the bottom panel of the figure, participant mean blink *reaction times* to target are plotted by target ISI. Blink reaction times roughly approximated the half-way point of the ISI, with a participant mean of 275.2 ms ± *standard deviation* = 101.0 for 500 ms ISI, 404.4 ms ± 120.2 [750 ms ISI], 510.7 ms ± 136.7 [1,000 ms ISI], 567.5 ms ± 142.4 [1,250 ms ISI], and 698.5 ms ± 198.7 [1,500 ms ISI].

A two-way ANOVA tested the effect of group x ISI on blink rate. A main effect of participant group x blink rate was found (*p* < 0.001; Figure 4), however, there was no effect of ISI condition (*p* = 0.053). Tukey post-hoc tests confirmed that ADHD/BPD participants had significantly higher blink rates than BPD (*p* = 0.002) and controls (*p* < 0.001). Blink rate did not vary among subjects when the timing of target appearance was random (*p* = 0.288). Given the main effect of blink rate in ADHD/BPD participants, we tested if blink rate in the metronome task interacted with impulsivity measures, which are related to ADHD symptomology. Indeed, blink rate positively correlated with ADHD/BPD participants’ total scores on the Barratt Impulsivity Scale (Rho correlation coefficient = 0.499; *p* = 0.021). Barratt Impulsivity subscale measures demonstrated positive trends with blink rate, such as Perseverance (Rho = 0.514; *p* = 0.017), Cognitive Complexity (Rho = 0.506; *p* = 0.019) and Attention (Rho = 0.436; *p* = 0.048), however, none of these results passed the Bonferroni correction for multiple comparisons (0.05/6 BIS subscales = 0.0083 corrected alpha).

**Figure 5:**
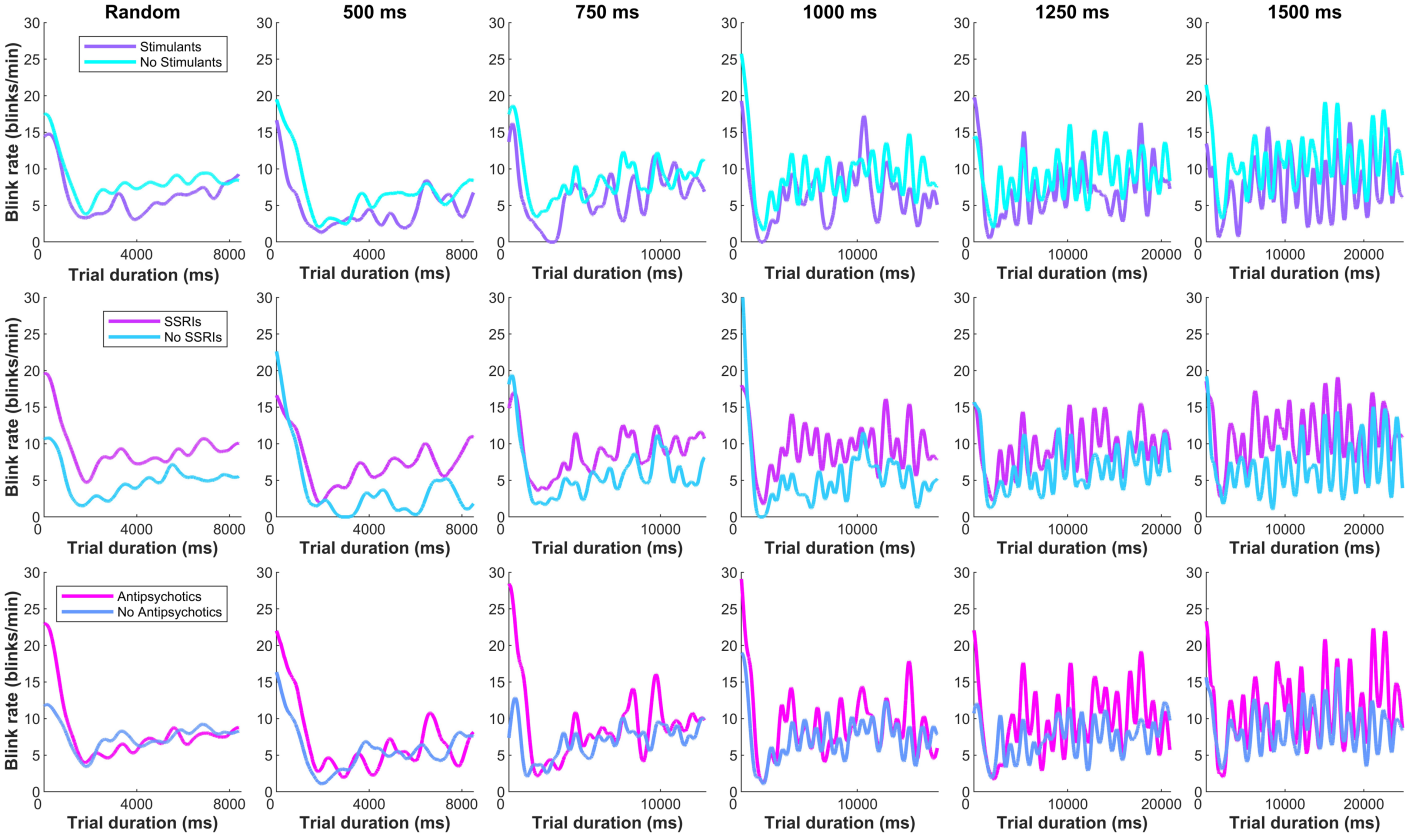
Moving average of clinical participants’ blink rate according to their psychotropic medication class prescription. No main effect of medication classes were observed on blink rates in the metronome task (averaged across the 5 ISI conditions): stimulant (*t*[42] = −0.89, *p* = 0.81), SSRI (*t*[42] = 1.48, *p* = 0.07), and second-generation antipsychotic (*t*[42] = 0.99, *p* = 0.16).

To understand if increased ADHD/BPD blink rates were in part driven by medication effects, one-tailed independent *t*-tests were performed according to whether clinical participants were taking any medications from three common psychotropic drug classes (SSRIs; psychostimulants; second-generation antipsychotics) for the metronome and random task, respectively. Blink rates were averaged across the 5 ISI conditions for metronome analysis, however, mean blink rates for each ISI are plotted in Figure 5. No main effect of medication classes on blink rates was observed in the metronome task: stimulant (*t*[42] = −0.89, *p* = 0.81), SSRI (*t*[42] = 1.48, *p* = 0.07), and SGA (*t*[42] = 0.99, *p* = 0.16). Nor was there a main effect of medication class on mean blink rates in the random task: stimulant (*t*[42] = −0.71, *p* = 0.76), SSRI (*t*[42] = 1.61, *p* = 0.057), and SGA (*t*[42] = 0.17, *p* = 0.43). Despite the lack of main effects of medication class on blink rate, Figure 5 reveals that there was a trend of increased blink rate for SSRIs versus no SSRIS and this was also observed in the random task as well (*p* = 0.07 and *p* = 0.05, respectively). It is possible that this trend may prove significant in a larger sample size or in experimental designs that apply ON versus OFF within-subject analysis. Based on these results, psychotropic medication prescriptions do not appear to be a significant contributor for the blink-related differences observed between ADHD/BPD participants versus BPD and controls.

#### 3.3.3. Blink timing relative to target appearance

To understand when blinks occurred, we evaluated the ‘reaction time’ of blinks relative to target appearance when targets were predictable (i.e., metronome task). A main effect of ISI on blink reaction time was found (*F* [4,331] = 82.908; *p* < 0.01) and this effect did not vary with group (*p* = 0.770). Blink reaction times steadily climbed with longer ISI durations (bottom panel of Figure 4), showing that participants blinked at the half-way point of the ISI. For example, in the 500 ms interval, average participants’ blink reaction time was 275 ms and in the 750 ms interval, average blink RT was 404 ms.

**Figure 6:**
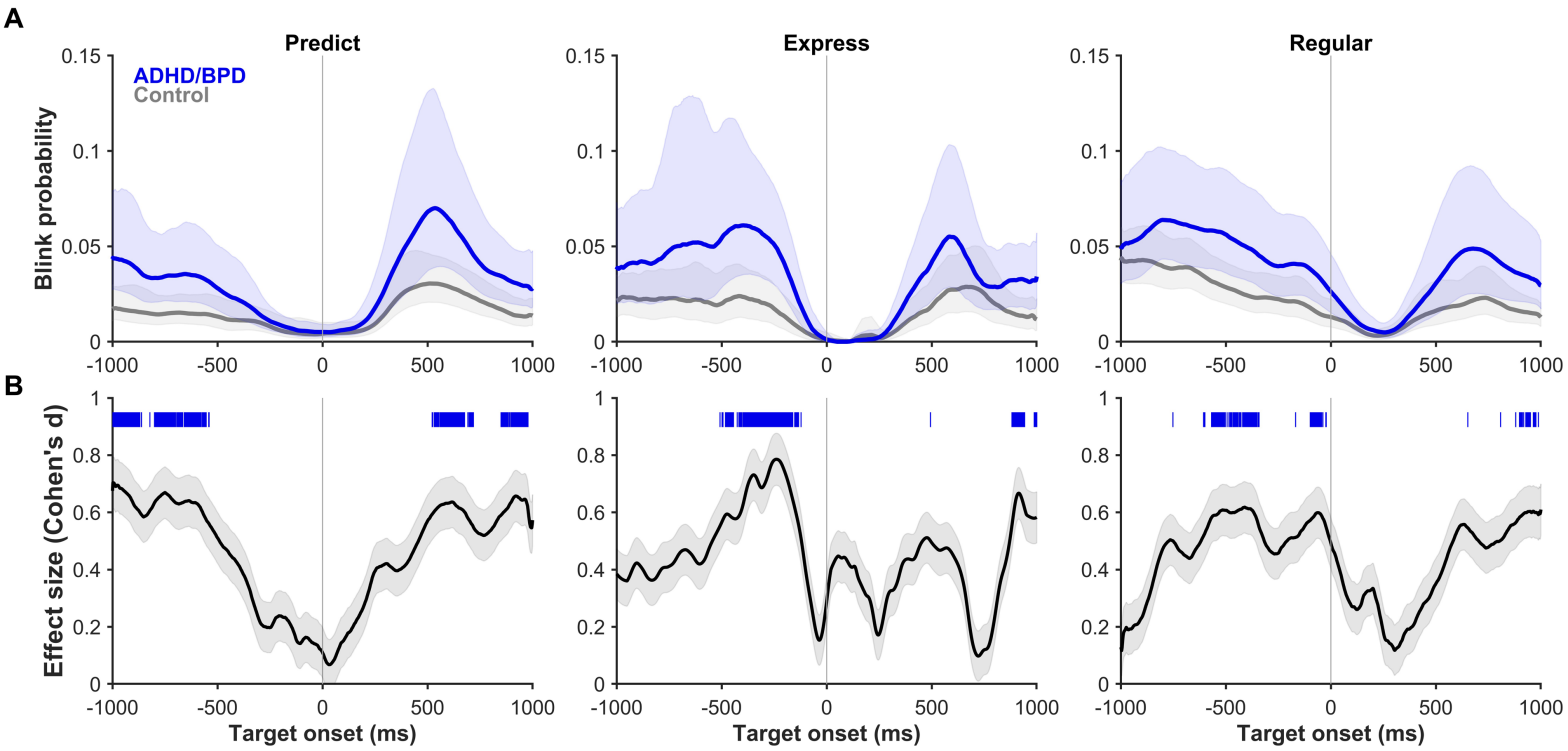
**(A)** Blink probability relative to target onset (vertical line at 0 ms) on metronome trials when ADHD/BPD participants (dark blue) and controls (gray) made a *predict* saccade (left panel), *express* saccade (middle panel), or *regular* saccade (right panel) to target. Shaded regions represent the 95% CIs of the individual mean blink probability averaged across subjects. **(B)** Effect sizes of the mean difference score of blink probabilities between ADHD/BPD and control groups relative to target onset. The absolute values of the Cohen’s d effect sizes are plotted with the shaded regions representing the 95% CI. Regions of statistically significant differences in blink probabilities between ADHD/BPD and control groups are highlighted as tick marks with the color corresponding to which group had a higher blink probability at that timepoint. As seen by the blue tick marks, ADHD/BPD participants had periods of significantly higher blink probabilities versus controls in predict, express and regular saccade trials.

Next, blink probabilities were analyzed on a trial-by-trial basis in the metronome task to determine if blink timing varied by saccade type. We sought to understand if the timing of blinks changed based on whether participants anticipated pacing targets or reflexively reacted to them. Mean blink probability and its 95% CI (shaded region) is plotted in Figure 6 for ADHD/BPD and control groups for the 1,000 ms before and after metronome target appearance. Periods where blink probabilities significantly differed by group are shown via significance lines at the top of panel B of Figure 6 with the color indicating which group had higher blink probabilities at that time. Standard Cohen d effect sizes for between-group differences at those time periods support large effect sizes between 0.6–0.8, with ADHD/BPD having higher blink probabilities than controls for all three saccade conditions.

The probability of blinks varied with saccade type relative to target onset at 0 ms (see vertical line). For both predict and express saccades, blink probability was near 0 at target onset for all three comparisons (see Supplementary Figure S4 for BPD versus controls and ADHD/BPD versus BPD), whereas it is about 0.02–0.04 for regular saccades. In the 200 ms epoch prior to target appearance, blink probability is near 0 for predict saccades, 0.02 for express saccades, and 0.04 for regular saccades, demonstrating a systemic change in likelihood of participants blinking relative to SRT. These data demonstrate that blink probabilities for 200 ms prior to target appearance, irrespective of group membership, can reasonably predict the type of saccade a participant is going to make to target. There was a main effect of group on blink probability (*F* [2,234] = 6.778; *p* = 0.01). In general, ADHD/BPD participants had the highest blink probabilities compared to BPD and controls. In sum, these data suggest that the timing of when a blink occurs is tightly coupled with saccade onset, which does not change with psychopathology, likely reflective of low-level midbrain-brainstem coordination, whereas the probability of blinks on a given trial indeed varies with psychopathology.

### 3.4. Pupil behavior

#### 3.4.1. Pupil size was elevated in BPD and ADHD/BPD

**Figure 7:**
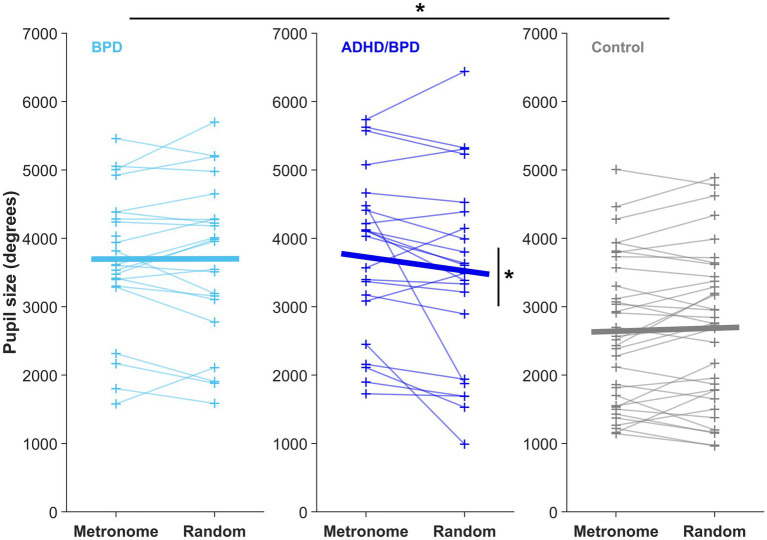
Mean pupil size from the time of saccade completion to 200-ms post-saccade in both experimental paradigms. Each + represents the mean pupil size of a subject. Horizontal bars display the group mean in the metronome and random tasks. There was a main effect of pupil size x participant group in both experimental tasks, *p < 0.05. Only ADHD/BPD participants showed a task-based pupil size effect, with an increased pupil size in the metronome versus random task (*Z* = −2.06, *p* = 0.039).

**Figure 8:**
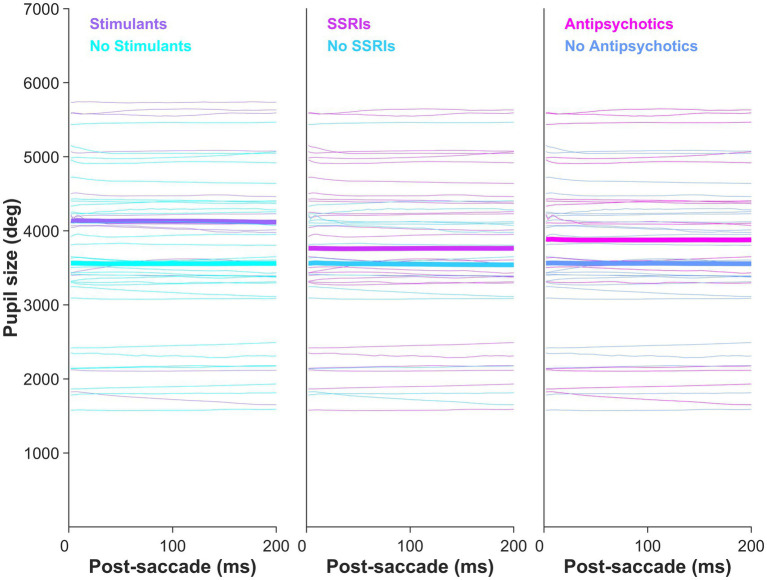
Clinical participants’ mean pupil size by psychotropic medication class prescription. Pupil size did not vary by psychotropic medication: stimulants (*p* = 0.122), SSRIs (*p* = 0.542), and second-generation antipsychotics (*p* = 0.339).

A main effect of pupil size by group was observed in the metronome task (*F* [2,76] = 9.78, *p* = 1.66e-04), with *post-hoc t*-tests revealing that pupil sizes of ADHD/BPD (*Mean* = 3,775.9 ± *SD* = 1,206) and BPD participants (3,696.2 ± 1,018) were significantly larger than controls (2,630.7 ± 1,083), *p* = 6.75e-04 and *p* < 6.21e-04, respectively (Figure 7). No difference in pupil size was found between ADHD/BPD and BPD participants (*p* = 0.997). A significant pupil size x group effect was also observed in the random task (*F* [2,75] = 5.2, *p* = 0.008), with significant *post-hoc* differences among ADHD/BPD and controls (*p* = 6.75e-04) and BPD and controls (*p* = 6.21e-04) (Figure 7). Further, there was a significant effect of task type (metronome versus random) on pupil size in ADHD/BPD participants, with a *Mean* of 3,775.91 ± *SD* = 1,205.97 in the metronome task and 3,473.61 ± *SD* = 1,433.00 in the random task (*Z* = −2.06, *p* = 0.039). No task-based effect was found on pupil size in BPD (*p* = 0.833) or controls (*p* = 0.293). There was no effect of pupil size x saccade type (*p* = 0.950), ISI of alternating targets (*p* = 0.998), or psychotropic medication class (stimulants [*p* = 0.122]; SSRIs [*p* = 0.542]; and SGAs [*p* = 0.339]) (Figure 8).

## 4. Discussion

In this study, we tested *temporal motor prediction* and *response inhibition* in pediatric patients with BPD and ADHD/BPD versus age- and gender-matched healthy controls by manipulating the predictability of the external environment and observing how this shaped motor action. When visual targets were predictable, BPD cohorts could successfully launch predictive saccades to anticipate the upcoming target and showed similar acquisition of learning to controls. However, when target appearances were random, the BPD with comorbid ADHD group launched significantly more anticipatory saccades than BPD and control groups, demonstrating a failure to wait for the cued target before initiating motor action. Compared to controls, participants with BPD and ADHD/BPD had larger pupil sizes in all task paradigms, evidence of increased sympathetic arousal. ADHD/BPD subjects were the only group to show a task-based modulation (i.e., metronome task versus random task) of blink and pupil parameters. When synchronizing movements to periodic targets, ADHD/BPD participants significantly increased their blink rate and pupil size, likely a reflection of increased neural effort. We discuss our findings in relation to previous work on temporal motor prediction and response inhibition in BPD and ADHD and their associated neural circuits.

### 4.1. Temporal prediction is normal in BPD with and without comorbid ADHD

Saccade main sequence did not differ among groups, supporting neurotypical saccade-related brainstem firing that produces the tight coupling between saccade amplitude and velocity. A previous eye-tracking study in ADHD also noted a normal saccade main sequence compared to control participants (Fried et al., 2014). Individuals with BPD or ADHD/BPD did not demonstrate an impairment in their ability to identify the rhythmicity of an alternating target and generate predictive saccades to match its arrival. On average, participants produced their first predictive saccade by the third target step (Supplementary Figure S2). In the 1,000 ms ISI condition, BPD participants outperformed control subjects by generating their first predictive saccade by the third target step instead of the fourth. Furthermore, no differences in the accuracy of predictive saccade timing with target appearance were observed among the three groups (Supplementary Figure S3). Generally, participants landed their saccades 100–200 ms before the stimulus arrival, allowing sufficient time for sensory processing of the visual stimulus (with a minimum afferent delay of approximately 50 ms) and endogenous initiation of the subsequent saccade (with a minimum efferent delay of approximately 20 ms) (Sparks, 1978; Fischer and Boch, 1983; Fischer and Ramsperger, 1984; Munoz and Wurtz, 1995; Schmolesky et al., 1998; White et al., 2017).

As expected, self-report measures of impulsivity in our study (Table 2) indicated significantly higher scores for individuals with BPD and ADHD/BPD compared to previously reported scores in neurotypical populations (Spinella, 2007). Despite the heightened impulsivity in the BPD and ADHD/BPD groups, their performance in temporal prediction was within the normal range. The predictability of visual targets and the allowance of fast motor actions may be well-suited for conditions favoring endogenous action instead of exogenous reaction. For instance, studies involving finger tapping to rhythmic visual stimuli in individuals with ADHD have shown no differences in mean synchronization times compared to controls (Rubia et al., 1999, 2001, 2003).

### 4.2. Increased anticipatory action in BPD with comorbid ADHD

We observed a main effect of participant group on the percentage of anticipatory saccades to unpredictable targets. Specifically, individuals with ADHD/BPD made twice as many anticipatory saccades as controls (Table 3), supporting a heightened waiting impulsivity. This finding is consistent with previous studies on cognitive control in ADHD, which reported increased anticipatory responses (Castellanos et al., 2000; Nigg et al., 2002; Feifel et al., 2004; Carr et al., 2006; Yep et al., 2018). This form of behavioral inhibition does not result from an automatic response to an uninhibited stimulus but rather represents a *disinhibition* of a motor response to an internal cue (Grootens et al., 2008).

The production of anticipatory saccades to targets has been largely attributed to dampened prefrontal cortex (PFC) signaling, which provides inhibitory input to saccade-related neurons in the superior colliculus to prevent a saccade motor command until information is present (Pierrot-deseilligny et al., 1991; Gaymard et al., 1998). The PFC sends various signals, including executive control over eye movements, one of which is motor suppression. In tasks requiring temporal delays before making a saccade to a specific spatial location, a subset of PFC neurons (e.g., frontal eye field and caudal PFC) has been shown to exhibit increased activity in non-human primates during the gap period, conveying ‘do not look’ signals (Hasegawa et al., 2004). Temporarily silencing these neurons resulted in monkeys making a saccade before the end of the gap period. Structural abnormalities and lesions of the PFC in humans have also been associated with reduced saccade suppression (Guitton et al., 1985; Walker et al., 1998; Pierrot-Deseilligny et al., 2003). Importantly, functional neuroimaging studies combined with motor suppression tasks consistently demonstrate abnormal PFC signaling in individuals with ADHD compared to controls (Feifel et al., 2004; Schneider et al., 2010; Hakvoort Schwerdtfeger et al., 2013). Therefore, we anticipate that the increased anticipatory saccades to random targets observed in individuals with BPD and ADHD correspond to abnormalities in PFC signaling.

Contrary to our results, two studies have reported increased anticipatory responses in individuals with BPD (Grootens et al., 2008; Parr et al., 2022). It remains unclear if this finding in BPD was partly driven by comorbid ADHD, as Grootens et al. (2008) did not exclude BPD participants with a comorbid ADHD diagnosis from study participation, nor did they report any potential differences in anticipatory responses between individuals with BPD and those with ADHD/BPD. Parr et al. (2022) in a study comparing anticipatory responses between pediatric participants with BPD and ADHD/BPD in a competitive mixed-strategy decision-making task, found no difference. However, the number of BPD participants with comorbid ADHD was limited (*n* = 12), and anticipatory responses were rare (i.e., 6.7% of trials in BPD), leading to statistical constraints in the BPD versus ADHD/BPD analysis (Parr et al., 2022). Previous associations between response inhibition and BPD symptomology have been explained by comorbid ADHD diagnoses (Nigg et al., 2005; Lampe et al., 2007). Based on our findings and previous data, we postulate that impaired response inhibition previously observed in performance tasks utilizing emotionally neutral stimuli in BPD could be partially explained by comorbid ADHD. Importantly, while self-report measures of impulsivity assessed by the Barratt Impulsivity Scale did not differ between individuals with BPD and those with ADHD/BPD (Table 2), we observed differences in behavior among BPD cohorts. These findings suggest an improved sensitivity in detecting impulsive behavior when using motor tasks rather than relying solely on self-report measures of symptomatology.

### 4.3. Blink modulation by group and temporal predictability

Blink rates did not differ among groups in the random task when participants were asked to reflexively react to upcoming targets or in the fixation epoch prior to metronome task start when participants were instructed to look straight ahead (dashed vertical lines in Figure 4). These task periods likely reflect baseline blink rate when behavior is untaxed by cognitive demands. This result is in keeping with previous results of no difference in blink parameters among children with ADHD and controls during rest (Daugherty et al., 1993; Groen et al., 2017). However, when participants synchronized their saccades in time with the alternating target, a main effect of group on blink rate was observed. ADHD/BPD participants had higher blink rates than BPD and control groups in the metronome task, perhaps due to the added attentional demand to maintain the interstimulus interval. This interpretation is consistent with our finding of an increased pupil size in ADHD/BPD participants in the metronome versus random task.

Further, this result is in agreement with a previous study showing differences in blink rate in ADHD versus controls during cognitive tasks (Fried et al., 2014). Fried et al. (2014) found that unmedicated adults with ADHD had higher blink rates than controls during a saccade task and failed to suppress blinks during periods of stimulus presentation. After administering their prescribed methylphenidate, the same participants underwent re-testing, revealing a decrease in their blink rate; however, it remained significantly higher than that of the control group. Moreover, medicated participants with ADHD exhibited greater suppression of blinks during intervals relevant to task performance. Unlike Fried et al.’s results, we did not observe differences in the timing of when blinks occurred relative to target onset in our ADHD/BPD group compared to BPD and controls (see Figure 6; Supplementary Figure S4), rather an increased frequency of blinks throughout the trial.

### 4.4. Blink rate and psychotropic medications

A limitation of our study is the absence of two data acquisition time points for comparing blink rates under medication ON and OFF conditions within the same subjects. However, it is worth noting that BPD patients are often treated with polypharmacy (Riffer et al., 2019), and we observed this pattern in our participants. Among our clinical participants, twenty-two were prescribed a medication from one psychotropic class, fifteen from two classes, four from three classes, and only three were not taking any psychotropic drugs. Therefore the opportunity for straightforward medication ON versus OFF analysis was limited. To examine whether the main effects of blink rates by group were influenced by medication classes, we compared the mean blink rates of clinical subjects based on their prescribed medication class. We found no significant difference in blink rate across medication classes, although there was a trend toward increased blink rates among those prescribed SSRIs (Figure 5). Previous research has shown that the administration of a strong centrally acting anticholinergic (promethazine hydrochloride) to controls can lead to an increased blink rate during a cognitive task paradigm, but this effect was not observed at rest (Naicker et al., 2016). Notably, metabolic modulation of the median raphe nucleus, which gives rise to most ascending serotonergic 5-HT projections to the cortex, has been shown to precede blinks in humans (Demiral et al., 2023). Within the ascending arousal network, modulation of BOLD activity in the median raphe nucleus prior to an eyeblink was second only to the substantia nigra, a site of dopamine synthesis (Demiral et al., 2023). Dopamine has been proposed to influence spontaneous blink rate through the inhibition of the spinal trigeminal complex via the basal ganglia’s inputs to nucleus raphe magnus and superior colliculus, causing increased frequency of spontaneous blinking (Kaminer et al., 2011). Serotonin has been previously implicated in the production of blinks through its innervation of the orbicularis oculi muscle (LeDoux et al., 1998) and its role within the sleep–wake cycle (Monti, 2011). Future research is encouraged to investigate whether SSRI drugs, known to induce adverse eye-related side effects (e.g., dry eye) (Koçer et al., 2015), also increase blink rates in users.

Our failure to observe a difference in mean blink rate among participants taking stimulant or antipsychotic medications is not surprising given the heterogeneity of the literature. Among the three studies that investigated blink rate in individuals with ADHD ON and OFF methylphenidate, only one reported a decrease in blink rate during a cognitive task in the ON condition, and the blink rate remained significantly higher than that of the control group (Daugherty et al., 1993; Fried et al., 2014; Groen et al., 2017). In another study, no relationship was found between PET measures of striatal dopaminergic receptor 1 and 2 availability and spontaneous blink rate in healthy adults (Demiral et al., 2022). Only under conditions of enhanced dopamine signaling, through the administration of a high dose of methylphenidate, was a relationship observed between putamen’s D1R availability and blink rate (Demiral et al., 2022). The authors attributed these results to a reserve of dopamine receptors at baseline (Nielsen and Andersen, 1992; Tadori et al., 2009; Covey et al., 2013), requiring either large-scale damage to dopaminergic structures (e.g., substantia nigra in Parkinson’s disease) or excessive receptor blockade (e.g., >80% D2R blockade for antipsychotic efficacy) for significant changes in blink rate. The dopamine receptor reserve theory postulates that low levels of dopamine signals can maintain normal function while excessive signaling can trigger pathological responses. Based on our findings of increased blink rates solely in the comorbid ADHD/BPD group, it is possible that the dopamine system is particularly stimulated in ADHD.

### 4.5. Increased pupil size in BPD

Pupil size is modulated by multiple processes, including global luminance (i.e., pupillary light reflex), orienting responses, cognition, stimulus salience, and autonomic tone through sympathetic and parasympathetic innervation (Loewenfeld, 1999; Alnæs et al., 2014; Wang et al., 2014). In our study paradigm, we controlled for global luminance and stimulus salience, with the only difference between tasks being the temporal properties of target arrival. Therefore, we interpret differences in pupillary behavior in terms of cognitive effort and autonomic tone. Pupil sizes following a saccade to the target significantly differed between groups in both the metronome and random tasks. The BPD and ADHD/BPD groups exhibited significantly larger pupil sizes than the control group in both tasks (Figure 8). Given that these results were observed across task sets with varying levels of difficulty, we propose that BPD patients exhibit elevated sympathetic tone compared to controls. Replication of these findings in future studies is needed due to its novelty. Interestingly, previous studies that examined pupil responses in ADHD participants during eye-tracking tasks reported either normal pupil sizes (Fried et al., 2014) or diminished sizes (Wainstein et al., 2017). Therefore, the finding of increased pupil sizes in our clinical participants may be specific to BPD pathology.

Like the blink rate findings, the ADHD/BPD group was the only group that showed a main effect of task condition on pupil size. Pupil sizes significantly increased in the metronome task compared to the random task for ADHD/BPD participants, likely indicating the increased cognitive load of the metronome task. In the pro- and anti-saccade task, pupil size increases during preparation to complete an anti-saccade and pupil size can reliably distinguish between correct and erroneous anti-saccade trials (Karatekin et al., 2010; Wang et al., 2015). In a visuo-spatial working memory task, pupil size corresponded with better task performance in ADHD and correlated with reaction time variability (Wainstein et al., 2017). Among clinical participants prescribed psychotropic medications, we observed that pupil size did not significantly differ based on medication class (Supplementary Figure S8). However, there was a trend toward increased pupil sizes in individuals prescribed stimulant medications, consistent with the findings of Wainstein et al. (2017).

### 4.6. Limitations

Our study has several limitations. Firstly, we did not ask pediatric psychiatry participants to interrupt their psychotropic medication schedule. Considering that some of the medications taken by our participants require extended washout periods and cannot be easily paused and resumed without the risk of complications, we made a deliberate decision to prioritize the well-being of our participants and refrain from interrupting their medication regimen. Nonetheless, it is important to acknowledge that medication effects may have potentially masked certain behavioral differences among the groups. To address this concern, we examined whether the prescribed medications accounted for the variability observed in the participants’ behavior. Our findings revealed that medications did not have a significant impact on the results of both blink and pupil parameters. However, further investigation involving testing under ON versus OFF medication conditions is recommended.

A second limitation is that we exclusively recruited female participants, so it is unknown whether males with BPD and ADHD/BPD would exhibit similar predictive saccade performance and eye-tracking metrics. Previous research supports gender-based differences in symptom presentation and brain circuitry in BPD, supporting the separate analysis of male and female BPD participants (McCormick et al., 2007; Herpertz et al., 2017). Furthermore, blink rate is known to differ between males and females (Bentivoglio et al., 1997; Sforza et al., 2008; Coors et al., 2021), highlighting the importance of analyzing blink parameters according to gender. However, based on large-scale eye-tracking studies, we do not expect saccade or pupil parameters to differ between females and males with BPD, as gender differences in these parameters have not been consistently reported across the lifespan (Hess and Polt, 1960; Luna et al., 2008; Tekin et al., 2018; Cascone et al., 2020; Yep et al., 2022).

Furthermore, we did not include an ADHD-only cohort to help determine whether the behaviors observed in BPD participants with comorbid ADHD were specifically related to ADHD or shared with BPD pathology. Future research in pediatric ADHD samples is needed to test whether our results of increased blink rate and pupil size in ADHD/BPD during tasks that tax cognitive demand is replicated.

## Data availability statement

The original contributions presented in the study are included in the article/Supplementary material, further inquiries can be directed to the corresponding author.

## Ethics statement

The studies involving human participants were reviewed and approved by Queen’s University Faculty of Health Sciences. Written informed consent to participate in this study was provided by participants aged ≥ 17 years old. Participants aged 16 years or below provided their oral assent and written consent was obtained by participants’ legal guardian/next of kin.

## Author contributions

OC, DB, LB, SK-K, and DM designed the experimental protocol. OC, AP, and SK-K recruited clinical participants. OC and AP recruited control participants, performed research and eye-tracking data collection. OC, DB, JH, IP, and BC wrote custom code and analyzed eye-tracking data. OC, AP, and DM wrote the manuscript. All authors provided manuscript edits and approved the submitted version.

## Funding

This work was supported by the Southeastern Ontario Academic Medical Organization AFP Innovation Fund Award SEA-17-004 to OC, LB, DM, and SK-K and the Canadian Institutes of Health Research Grant MOP-FDN-148418 to DM. OC is supported by an Ontario Graduate Scholarship. DM is supported by the Canada Research Chair Program.

## Conflict of interest

The authors declare that the research was conducted in the absence of any commercial or financial relationships that could be construed as a potential conflict of interest.

## Publisher’s note

All claims expressed in this article are solely those of the authors and do not necessarily represent those of their affiliated organizations, or those of the publisher, the editors and the reviewers. Any product that may be evaluated in this article, or claim that may be made by its manufacturer, is not guaranteed or endorsed by the publisher.

## Supplementary material

### Supplementary FIGURE 1

Main sequence of predict, express, and regular saccades to metronome targets by subject group. Square root model fits for peak saccade velocity x amplitude were computed for each individual participant and averaged across participants. Model coefficients are reported with 95% CI values listed in parenthesis. The increased frequency of regular saccades with 10° amplitudes represent saccades to the first step 10° away from central fixation point, whereas target steps 2–16 were 20° in separation. Main sequence model fits did not vary by participant group in any of the three saccade types.

### Supplementary FIGURE 2

Averaged saccade reaction times to metronome targets at 5 different ISIs and to unpredictable targets in the random ISI condition. Square markers represent the mean SRT at the target step according to group membership. Lines connect the mean SRTs as a function of target sequence in the experimental trial. Errors bars show mean ± standard deviation. Horizontal SRT line at 90 ms denotes the separation between predictive and reactive saccades. Predict saccades were on average launched to metronome target by target steps 3-4. ADHD/BPD participants’ mean SRT to target steps 2-16 in the random task show non-visually guided saccades toward temporally unpredictable targets.

### Supplementary FIGURE 3

Accuracy of predict saccades depicted by the end of the saccade relative to target appearance (‘end SRT’; vertical line at 0 ms). Histograms show kernel density estimates for predict saccades (SRT<90 ms) made by participants (ADHD/BPD in dark blue, BPD in light blue, controls in gray) to the 5 metronome targets conditions and 1 randomized ISI condition. Participants tended to end their saccades 100-200 ms before the target appeared and this distribution became broader with longer ISI durations as well as with target unpredictability. No difference was observed in temporal accuracy x subject group.

### Supplementary FIGURE 4

**(A)** Blink probability relative to target onset (vertical line at 0 ms) on metronome trials when BPD participants (dark blue) and controls (gray) made a *predict* saccade (left panel), *express* saccade (middle panel), or *regular* saccade (right panel) to target. Shaded regions represent the 95% CIs of the individual mean blink probability averaged across subjects. **(B)** Effect sizes of the mean difference score of blink probabilities between BPD and control groups relative to target onset. The absolute values of the Cohen’s d effect sizes are plotted with the shaded regions representing the 95% CI. Regions of statistically significant differences in blink probabilities between BPD and control groups are highlighted as tick marks with the color corresponding to which group had a higher blink probability at that timepoint. There are brief periods when BPD participants had significantly higher blink probabilities than controls during express and regular saccade trials (see light blue tick marks), however, this occurs for <100 consecutive ms. **(C)** and **(D)** reflect the same analysis for ADHD/BPD versus BPD participants.
