# Supplementary figures and images for: Motor synchronization and impulsivity in pediatric borderline personality disorder with and without attention-deficit hyperactivity disorder: an eye-tracking study of saccade, blink and pupil behavior
Source: Front Neurosci. 2023 Jun 22;17:1179765. doi: 10.3389/fnins.2023.1179765 (PMC10323365; doi:10.3389/fnins.2023.1179765)

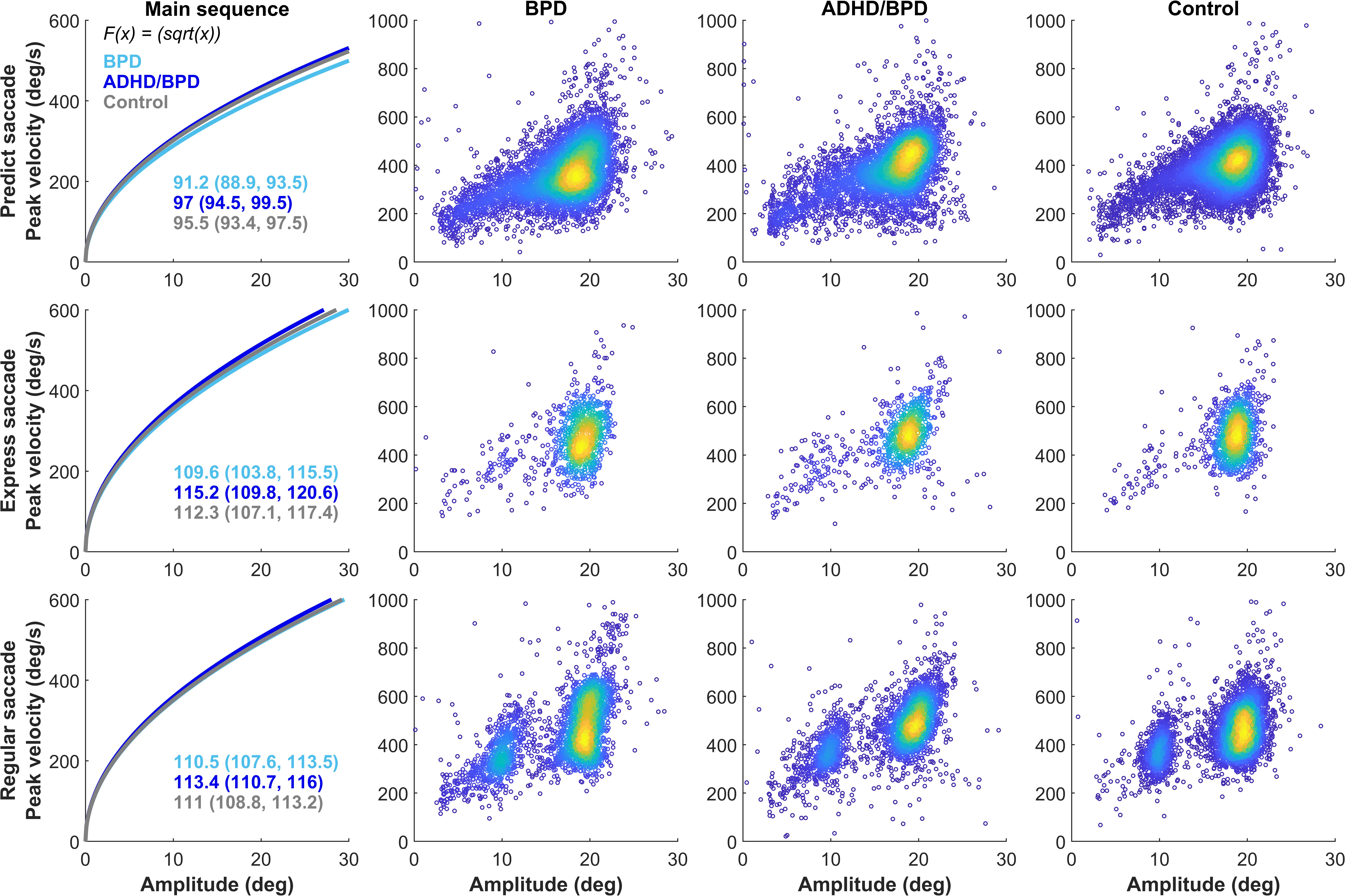

Supplement: Supplementary file 1 [file Image_1.JPEG]

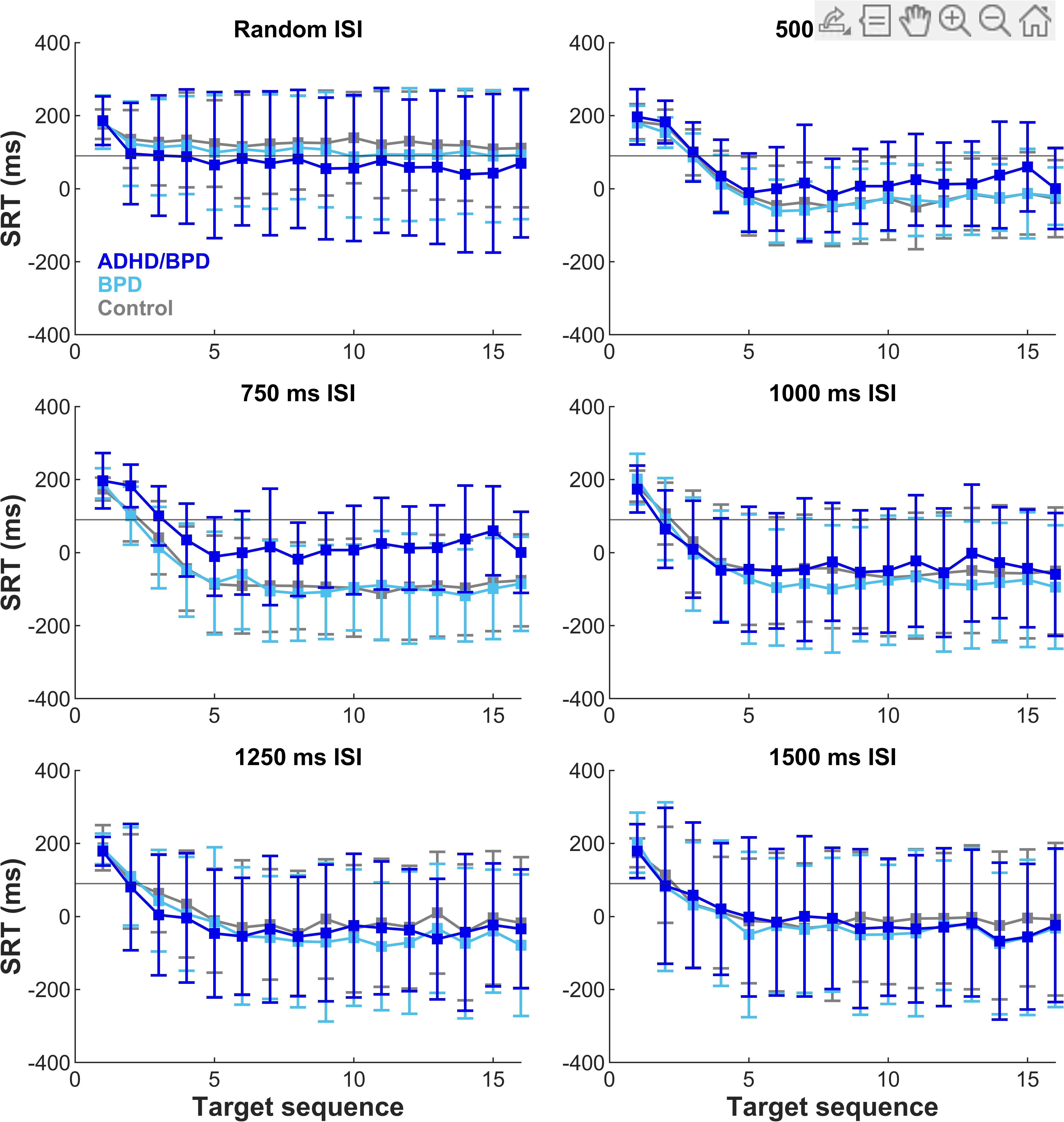

Supplement: Supplementary file 2 [file Image_2.JPEG]

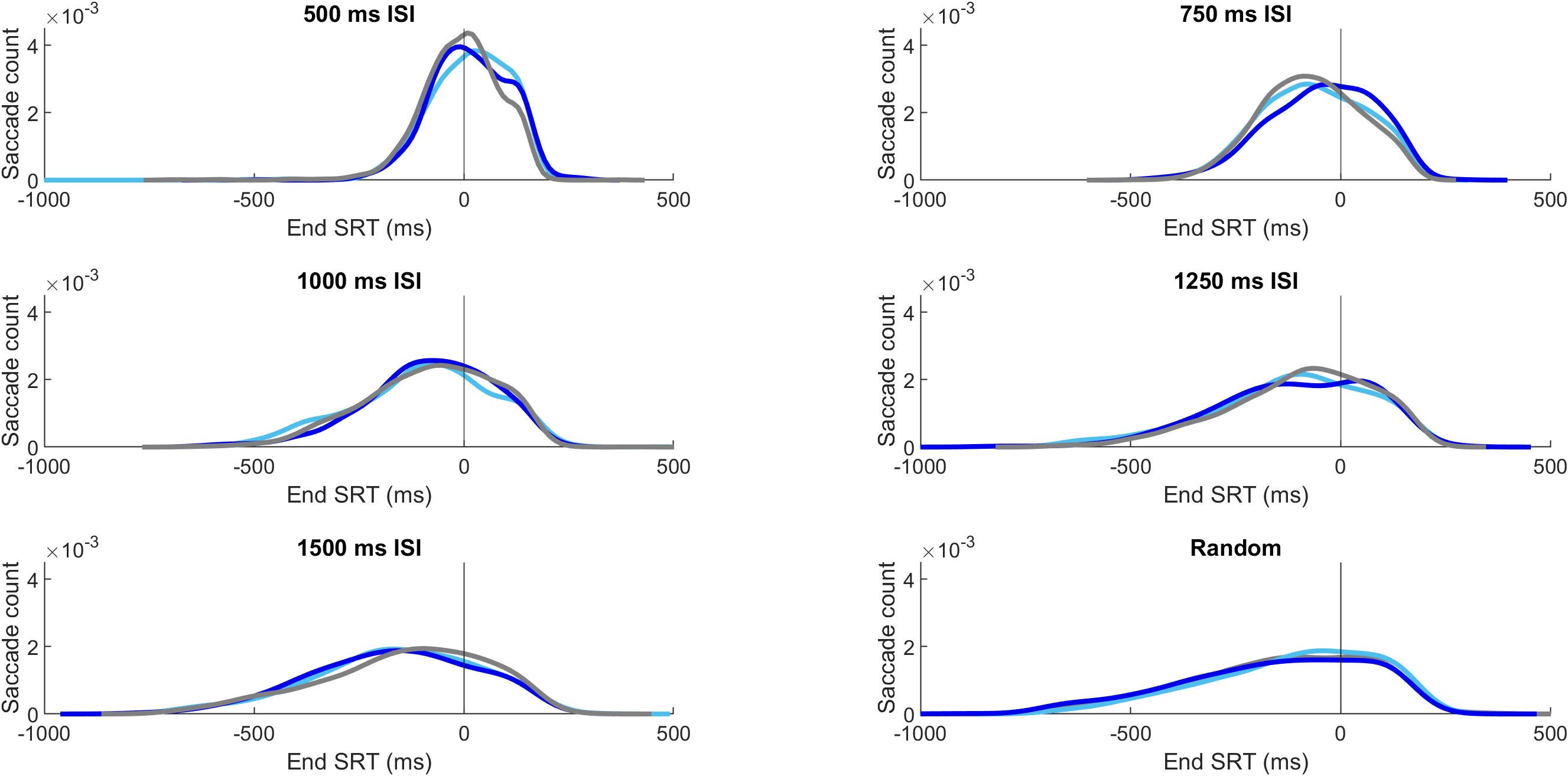

Supplement: Supplementary file 3 [file Image_3.JPEG]

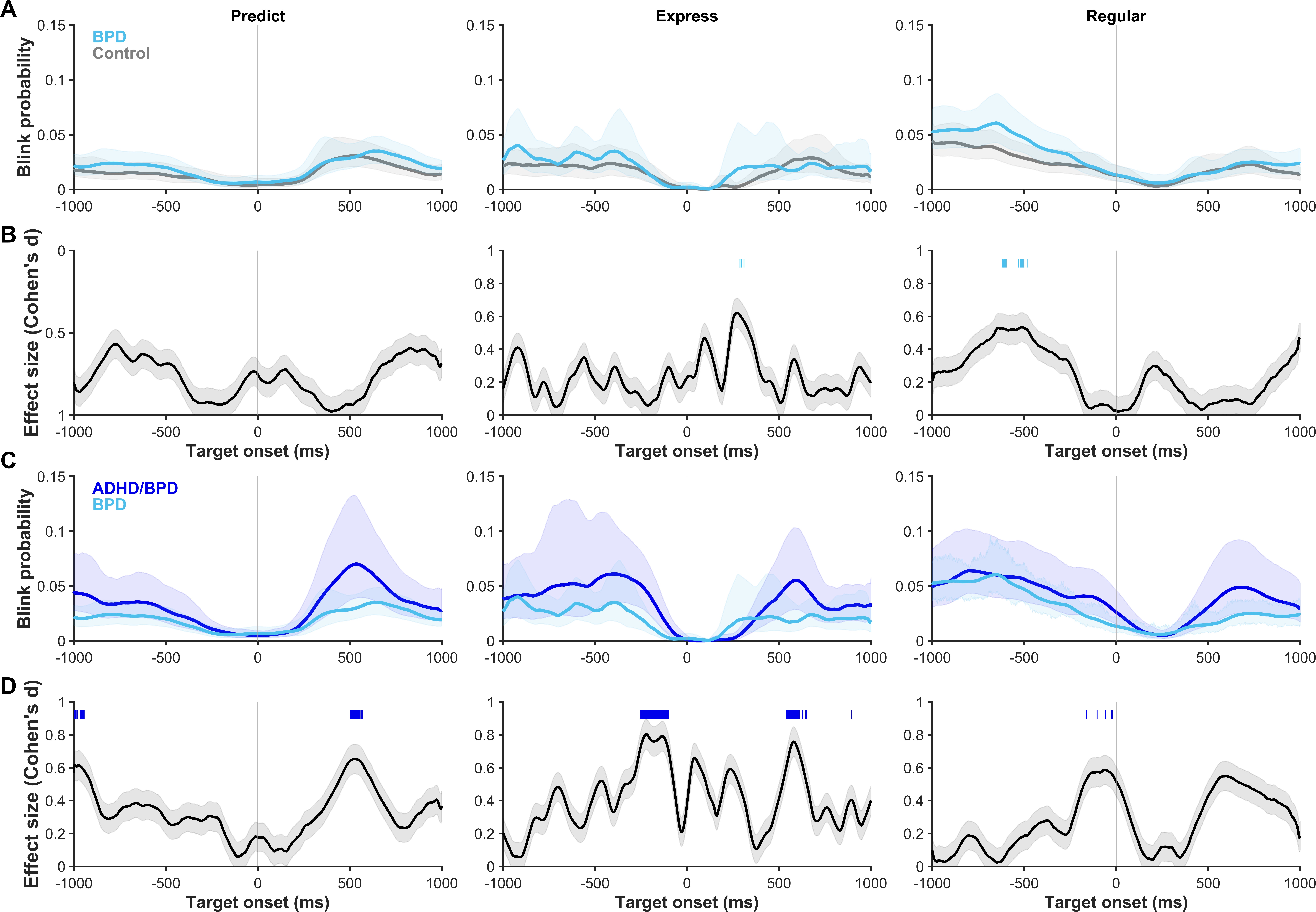

Supplement: Supplementary file 4 [file Image_4.JPEG]
